# Proteasome- and Ethanol-Dependent Regulation of HCV-Infection Pathogenesis

**DOI:** 10.3390/biom4040885

**Published:** 2014-09-29

**Authors:** Natalia A. Osna, Murali Ganesan, Terrence M. Donohue

**Affiliations:** 1 Research Service, Veterans Affairs Nebraska-Western Iowa Health Care System, 4101 Woolworth Ave, Omaha, NE 68105, USA; E-Mails: murali.ganesan@unmc.edu, (M.G.); tdonohue@unmc.edu (T.M.D.Jr.); 2 Department of Internal Medicine, University of Nebraska Medical Center, Omaha, NE 68105, USA; 3 Department of Biochemistry and Molecular Biology, University of Nebraska Medical Center, Omaha, NE 68198, USA

**Keywords:** hepatitis C virus, proteasome, PA28, interferon-sensitive genes, ubiquitylation, antigen presentation, ethanol

## Abstract

This paper reviews the role of the catabolism of HCV and signaling proteins in HCV protection and the involvement of ethanol in HCV-proteasome interactions. HCV specifically infects hepatocytes, and intracellularly expressed HCV proteins generate oxidative stress, which is further exacerbated by heavy drinking. The proteasome is the principal proteolytic system in cells, and its activity is sensitive to the level of cellular oxidative stress. Not only host proteins, but some HCV proteins are degraded by the proteasome, which, in turn, controls HCV propagation and is crucial for the elimination of the virus. Ubiquitylation of HCV proteins usually leads to the prevention of HCV propagation, while accumulation of undegraded viral proteins in the nuclear compartment exacerbates infection pathogenesis. Proteasome activity also regulates both innate and adaptive immunity in HCV-infected cells. In addition, the proteasome/immunoproteasome is activated by interferons, which also induce “early” and “late” interferon-sensitive genes (ISGs) with anti-viral properties. Cleaving viral proteins to peptides in professional immune antigen presenting cells and infected (“target”) hepatocytes that express the MHC class I-antigenic peptide complex, the proteasome regulates the clearance of infected hepatocytes by the immune system. Alcohol exposure prevents peptide cleavage by generating metabolites that impair proteasome activity, thereby providing escape mechanisms that interfere with efficient viral clearance to promote the persistence of HCV-infection.

## 1. HCV, the Proteasome and Oxidative Stress

Approximately 170 million individuals worldwide and nearly 5% of all adult Americans aged 46–64 are infected with hepatitis C. Chronic viral hepatitis is a potential risk for cirrhotic liver disease and life-threatening complications of portal hypertension and hepatocellular carcinoma [1]. Between 1990 and 2005, the prevalence and number of people with HCV antibodies in their circulation increased from 2.3% to 2.8% of the world population and from >122 million to >185 million. Central and East Asia and North Africa/The Middle East are estimated to have the highest prevalence (>3.5%). However, South and Southeast Asia, Sub-Saharan Africa, Central and Southern Latin America, the Caribbean, Oceania, Australia and Central, Eastern and Western Europe have moderate prevalence (1.5%–3.5%); whereas Asia Pacific, Tropical Latin America and North America have the lowest prevalence (<1.5%) [2]. Chronic HCV infection is often associated with lipid metabolism disorders, as HCV possesses lipophilic properties and is assembled on lipid droplets [3].

HCV is a member of the Flaviviridae family and is a single-stranded RNA virus. HCV RNA replication generates a 3000 amino acid polyprotein, which then is cleaved by the host signal peptidase, the signal peptide peptidase and the virus’s own HCV proteases, generating structural and non-structural proteins [4]. The structural region of HCV includes core-E1-E2-p7 proteins, while the non-structural region includes NS2, the NS3-4A complex, NS4B, NS5A and NS5B proteins. The expression of several HCV proteins, including core and NS5A, may cause the development of intracellular oxidative stress. *In vitro* cell culture experiments using CYP2E1- and HCV core-transfected cells have shown that the HCV core and NS5A proteins induce oxidative stress, which is further enhanced by the products of cytochrome P450 2E1 CYP2E1, one of the enzymes that catalyzes ethanol oxidation [5,6,7].

The function of some enzymes, including the proteasome, is tightly regulated by oxidative stress [8,9]. The proteasome is the predominant intracellular proteolytic enzyme. It exists in several forms: one form is the 26S particle (20S catalytic core and two 19S cap particles); another is the free proteolytically active 20S particle that is devoid of the cap particles; the third form is a combination of both particles (hybrid proteasome). The 26S proteasome degrades ubiquitylated proteins, whereas the 20S proteasome degrades non-ubiquitylated (often oxidatively modified) proteins. The 26S proteasome is more sensitive to oxidative stress than the 20S form of the enzyme, due to rapid dissociation of the 19S caps from the 20S catalytic core [10]. However, the activity of the 20S proteasome is regulated by the level of oxidative stress: low oxidative stress (specifically, peroxynitrite-induced) enhances proteasome activity, while high oxidative stress (including that induced by prolonged exposure to high doses of ethanol *in vivo*) suppresses proteasome activity [11,12,13].

The 20S proteasome structure consists of outer α-subunits and interior β-subunits in a cylinder-shaped arrangement. Alpha-subunits are responsible for the cylinder’s shape, while β-subunits (both constitutive and immunoproteasome (IPR)) catalyze proteolysis. The distribution of constitutive proteasome and IPR is tissue-specific. Thus, skeletal muscle is rich in constitutive proteasome β subunits, while immune cells contain high levels of IPR β subunits. However, in the liver, there is a mixture of the constitutive and IPR forms. Replacement of constitutive β subunits with IPR subunits is crucial for the maturation and cleavage of antigenic peptides for MHC class I-restricted antigen presentation [14,15,16]. One of the most important IPR subunits that cleaves antigenic peptide is LMP7 (aka β5i), which possesses a unique chymotrypsin-like (Cht-L) activity. The existence of a mixed (“intermediate”) proteasome with partial incorporation of IPR subunits broadens the variety of generated antigenic peptides that form a complex with MHC class I to be recognized by cytotoxic T-lymphocytes (CTL) on the hepatocyte surface [17].

Proteasome activity is also regulated by 19S caps on the 26S enzyme and by PA28 regulatory isoforms for the 20S proteasome. Specifically, PA28 α,β enhances the cytosolic 20S form of proteasome, and PA28γ activates the nuclear 20S enzyme. These regulators enlarge the opening of the 20S catalytic core, thereby enhancing access by substrate proteins to the catalytic centers. PA28 α,β activity (as well as replacement of the constitutive proteasome by the immunoproteasome) is elevated by interferons. Proteasome activity in this case is stimulated by IFN-initiated peroxynitrite release, because peroxynitrite dose-dependently modulates proteasome activity [13,18]. Cytoplasmic PA28α,β and nuclear PA28γ may both be important regulators of the proteasome’s ability to degrade oxidatively-damaged proteins, and these activators likely play a role in cell adaptation to oxidative stress [19,20].

HCV induces oxidative stress through multiple mechanisms. The HCV core protein binds to the outer mitochondrial membrane, thereby affecting mitochondria respiration [5,21]. HCV changes the levels of the mitochondrial chaperone, prohibitin, causing disruption of the mitochondrial respiratory chain and the overproduction of reactive oxygen species (ROS) [22]. The HCV core protein, along with NS5A and NS3 proteins, increases calcium uptake by mitochondria to suppress the levels of reduced mitochondrial glutathione, which then enhances ROS release [23]. However, the transcription factor, Nrf2, controls oxidative stress by elevating the elimination of oxidatively-damaged proteins. Specifically, it increases the expression of the 20S proteasome and the proteasome activator, PA28αβ. Activation of Nrf2 is induced by core, E1, E2, NS4B and NS5A HCV proteins; the HCV core protein is the most potent activator of Nrf2 (reviewed in [23]). Nrf2 activation by HCV is mediated by mitogen-activated protein kinase (MAPK) p38 [24]. Induction of the 20S proteasome activity by the core protein may be mediated by core-induced Nrf2 activation, as the core protein elevates 20S chymotrypsin-like activity directly, via core-proteasome interactions, and indirectly, by induction of oxidative stress. If oxidative stress reaches high levels (as in the case of HCV-infected liver cells exposure to ethanol and its metabolites), the 20S proteasome activity declines significantly [25].

## 2. The Proteasome-Dependent Regulation of HCV Propagation

HCV propagation is tightly regulated by the ability of intracellular protein-degrading systems to cleave HCV proteins. Most HCV proteins are degraded by the 26S proteasome following their ubiquitylation. Usually, activation (phosphorylation) of these viral proteins precedes ubiquitylation. As an example, HCV envelope protein, E2, which facilitates viral entry, localizes in the cytosol. After phosphorylation of E2 by protein kinase R, E2 is subsequent ubiquitylated and degraded [26]. Furthermore, NS2, a short-lived protein expressed in the context of HCV polyprotein, appears to be sensitive to proteasomal degradation that specifically targets phosphorylated NS2 proteins [27]. Furthermore, some polyubiquitylated non-structural HCV proteins, including NS5A and NS5B proteins, important components of the HCV replication complex, are also degraded by the 26S enzyme [28,29]. In addition to ubiquitylation, modification of NS5A with ubiquitin-like modifier, ISG15 (ISGylation) reduces of NS5A protein expression and its stability [30], which limits viral propagation. Furthermore, p7, an ion-channel protein that regulates the HCV life cycle, is degraded by the proteasome [31].

HCV proteins can also be degraded by the 20S form of the proteasome when they undergo post-translational modifications that render them susceptible to proteolysis without prior ubiquitylation. Other post-translational modifications of HCV proteins include glycosylation of HCV envelope proteins, methylation and acetylation of NS3 protein, biotinylation of NS4A protein and palmitoylation of core and NS4B proteins [32]. Although it is known that post-translational modifications change protein function/stability, the exact role of certain modifications in viral propagation is not yet clear.

Currently, the most significant progress in characterizing the roles of Ub-dependent and Ub-independent degradation pathways has been found with the HCV core protein. It appeared that the Ub-HCV core protein can be processed by the 26S proteasome in the cytosol, while the un-Ub-core is processed by the 20S enzyme in the nucleus to activate steatosis-regulating genes, which contribute to HCV pathogenesis. In the cytosol, the HCV core protein is ubiquitylated by E6AP, an E3 ubiquitin ligase, and then is degraded, thereby preventing viral propagation. A nuclear proteasome activator, PA28γ, is involved in Ub-independent proteasomal degradation of the core protein [33,34]. Knockdown of either E6AP or PA28γ stabilizes the core protein (reviewed by [35]). Importantly, exclusive knockdown of nuclear PA28γ enhances ubiquitylation of the core protein and slows virus production (but not HCV RNA replication), whereas knockdown of E6AP reduces ubiquitylation of the core protein and enhances virus production ([36,37,38]). Additionally, knockdown of PA28γ causes accumulation of un-ubiquitylated HCV core in the nucleus. Such knockdown disrupts steatosis/enhancement of fatty acid biosynthesis [39,40], while upregulation (by overexpression) of nuclear PA28γ is related to hepatocellular carcinoma development [41]. Thus, PA28γ plays a crucial role in the development of liver pathology induced by HCV-infection.

## 3. The Proteasome and HCV-Dependent Evasion of Viral Clearance

The ability of the proteasome to degrade both ubiquitylated and non-ubiquitylated viral and signal transduction factors is an important part of HCV-infection pathogenesis. To cleave these proteins, the proteasome must be catalytically active. Such activity is regulated through the cytokine network. Interferons (both type 1 and type 2) are among the most potent proteasome/immunoproteasome activators. Thus, HCV-modified interferon production, as well as HCV-impaired transduction of interferon signal in infected liver cells may affect proteasome function, which, in turn, is crucial for HCV propagation and proteasome-dependent immune reactions.

Viral infection initiates a complex series of “defense” events that protect cells from further infection by activating intracellular anti-viral systems to prevent the spread of virus to neighboring cells. These latter events are encoded by interferon (IFN)-sensitive genes (ISGs). The host response is developed when a pathogen-associated molecular pattern (PAMP) presented by the infecting virus is recognized and engaged by specific PAMP receptors, which initiate signals that induce the expression of antiviral effector genes. In HCV-infected hepatocytes, this recognition works through toll-like receptors (TLRs). TLR3 plays the major role of “sensing” RNA viruses, including HCV. Intracellularly, HCV can be also recognized by the intracellular receptor, retinoic-acid-inducible gene I (RIG-I). Engagement of these receptors/pathways finally causes activation of interferon-regulating factors (IRFs), specifically, IRF3 phosphorylation, which is necessary for the activation of early ISGs and IFNβ-production. As the next step of anti-viral protection, there is initiation of IFN type 1 signaling through the Jak-STAT1 pathway that activates multiple anti-viral “late” ISGs and is responsible for IFNα production (reviewed in [42]). By itself, IFN possesses no anti-viral activity, but through its activation of “early” and “late” ISGs, IFN initiates multi-targeted antiviral effects in infected cells.

Actually, HCV is an inefficient activator of anti-viral protective pathways. The virus downregulates “early” and “late” ISGs, which delays or even prevents subsequent HCV clearance. These latter events are believed to require interplay between HCV and the ubiquitin-proteasome system (UPS) [35]. Activation of innate immunity to viruses involves PAMP-recognizing receptors, such as RIG-I, which senses viral RNA and triggers a signaling pathway that also recruits the outer mitochondrial membrane “adaptor” protein, mitochondrial antiviral signaling (MAVS). Activation of this pathway requires phosphorylation of IRF3 followed by activation of “early” ISG. To initiate this signaling, RIG1 and MAVS are ubiquitylated by the E3 ligase, Trim25 [43,44]. In addition, Riplet E3 ligase mediates K64-linked polyubiquitylation of RIG1 [45]. However, as an alternative to ubiquitylation, HCV induces ISGylation of RIG1 by a small ubiquitin-like modifier, ISG15, which prevents RIG1 ubiquitylation [46], thereby blocking RIG1-induced signaling. In addition, HCV protease, NS3/NS4, cleaves an adaptor protein MAVS, which hampers the protective innate immunity response [47]. Thus, ubiquitylation of the signaling proteins involved in ISG activation is a prerequisite for anti-viral cell protection through innate immunity mechanisms. In HCV-infected cells, it is not robustly activated, making hepatocytes very susceptible to further HCV-infection.

In addition to suppressing “early” ISGs, HCV also disrupts the activation of “late” ISGs by blocking IFN signaling through the Jak-STAT1 pathway. The virus suppresses STAT1 signaling by degrading ubiquitylated STAT1 [48], as well as IFNα-activated STAT3 [49]. At the downstream level of Jak-STAT1 signaling, HCV induces protein phosphatase, PP2A, which blocks arginine protein methyltransferase 1 (PRMT1)-mediated methylation of STAT1 necessary for the attachment of phosphorylated STAT1 to DNA [50,51,52]. Impairment of IFN-induced signaling through the Jak-STAT1 pathway also negatively regulates the proteasome activation/IPR expression, which is modulated through this pathway. As already mentioned, IFN is the most potent proteasome activator.

Other IFN-dependent factors activated by the Jak-STAT1 pathway are involved in the antigen presentation of viral peptides [53,54]. For efficient clearance of HCV from infected cells, clonally expanded, antigen-specific cytotoxic T-lymphocytes (CTLs) recognize virally-modified MHC class I on the cell surface. HCV can evade the immune response by interfering with its activation and presentation of MHC class I-peptide targeted complexes on infected cells to CTLs. However, HCV not only affects such presentation on hepatocytes, but it also impairs the antigen-presentation machinery on the dendritic cells [55]. Thus, the impairment of the HCV-infected cell clearance partially depends on the proteasome function in both immune cells and target hepatocytes.

In HCV-infected hepatocytes, IPR is the principal form of the proteasome that cleaves viral proteins to generate suitably-sized peptides for antigen presentation. The high frequency of mutations within the HCV genome can change the proteasome cleavage and impose limits to MHC class I-restricted presentation [56,57]. Other factors involved with antigenic peptide generation relate to IPR function itself. Thus, HCV protease NS3 suppresses the IPR subunit, LMP7, which is crucial for chymotrypsin-like peptidase activity in the sub-genomic HCV replicon. This interaction between NS3 and IPR is direct, and it ultimately causes impaired antigenic peptide generation [58], which partially explains why LMP7 polymorphisms in HCV-infection are linked to disease outcomes [59]. We found that the combination of HCV and ethanol strongly inhibits the proteasome function and subsequent antigenic peptide cleavage and presentation in transgenic (Tg) HCV mice that express HCV structural proteins. These effects are partially dependent on ethanol/HCV-induced oxidative stress [12,60] and ethanol-induced proteasome methylation status, which also regulates proteasome activity [61].

When we isolated the 26S proteasome from livers of HCV core-Tg mice that were fed a control or ethanol liquid diet, ethanol feeding suppressed the levels of Ecm29, a protein that regulates the 26S proteasome stability and coupling to intracellular secretory compartments engaged in quality control [62,63,64]. The observed reduction in 20S content within the 26S enzyme in livers of HCV core-Tg alcohol-fed mice is likely a consequence of the proteasome instability. Thus, ethanol feeding destabilizes the 26S proteasome, which may lead to partial HCV-associated depletion of the 20S particle in the 26S enzyme, which may decrease the efficiency of ubiquitylated protein degradation. Furthermore, ethanol-fed mice had lower levels of the 19S proteasome-associated deubiquitylase, UCHL5, than control mice. Furthermore, the content of PA28 α, an activator of the 20S (and hybrid 26S/20S) proteasome that binds to the α-subunits on the 20S enzyme to enhance the accessibility of substrate proteins to the interior of the proteolytic core, was decreased in the proteasome isolated from the livers of HCV^+^ mice and was further reduced in these mice after ethanol feeding. Finally, the aforementioned changes in the 26S proteasome from HCV-Tg mice fed ethanol caused impaired presentation of the peptide-MHC class I complex on the hepatocyte surface [12]. Interestingly, similar oxidative stress/protein methylation-dependent reduction in proteasome chymotrypsin-like activity was reported in ethanol-exposed NS5A-Tg mice [7]. Collectively, these experimental results suggest that in humans, the combination of HCV and ethanol exacerbates the clinical course of HCV-infection. This hypothesis is well supported by clinical data and by reports of numerous immune dysfunctions in HCV^+^ alcoholics [65,66].

## 4. Summary and Conclusions

Both the UPS and the free 20S proteasome are the predominant proteolytic pathways in eukaryotic cells. The foregoing review has described the anti-HCV function of the ubiquitin-proteasome system in hepatocytes. Clearly, ubiquitin-mediated proteolysis and degradation by the free 20S proteasome destroy several key HCV proteins, which include the HCV core protein, nonstructural proteins that participate in viral replication, viral entry proteins and the p7 ion channel protein. The most efficient way of preventing HCV propagation is the degradation of ubiquitylated HCV proteins (core protein) by the 26S proteasome. Furthermore, HCV antigen degradation by the IPR generates antigenic peptides for presentation on the surface of HCV-infected hepatocytes, resulting in the death and removal of these cells. To counter these anti-viral mechanisms, Hepatitis C virus has evolved escape strategies that prolong its ability to infect. One is its rapid rate of mutation, which causes variation in and thereby limits the numbers and types of antigenic peptides generated by the proteasome. Other subversion strategies include HCV proteases that degrade key proteasome subunits and activators (proteasome-associated proteins). HCV synergizes with alcohol in generating oxidative stress, which exacerbates liver injury. Alcohol consumption thwarts the antiviral potential of the proteasome, because its chronic metabolism in liver cells generates superoxide, acetaldehyde and nitric oxide, each of which can undergo secondary reactions to form other oxidants (e.g., peroxynitrite and malondialdehyde-acetaldehyde) that impede the proteasome activity. Such inhibition interferes with antigen presentation and viral elimination. Finally, the recent excitement over the success of a new generation of oral HCV medications, such as Sovaldi^®^ (sofosbuvir, Gilead Sciences, Foster City, CA, USA), which blocks viral replication, has greatly simplified treatment for HCV and has resulted in an unprecedented cure rate of infected patients [67]. Nevertheless, the latest findings reported here underscore the importance of maintaining (and seeking strategies to activate) the liver’s own UPS as an additional means of adaptive immunity, thereby blocking the infection and spread of hepatitis C virus.

In conclusion, the UPS plays a significant role in preventing HCV propagation and the proteasome-dependent regulation of anti-viral immune responses. Ethanol combined with HCV suppresses proteasome function, thereby providing escape mechanisms preventing efficient clearance of HCV-infected cells and promoting the persistence of HCV infection. Ethanol synergizes with HCV in suppressing proteasome function and subsequent proteasome-dependent events that limit the spread of virus among hepatocytes and eliminate HCV.

## Abbreviations

HCV: hepatitis C virus
NS5A: non-structural protein 5 A
IFN: interferon
CYP 2E1: cytochrome P450 2E1
IPR: immunoproteasome
LMP7: hepatitis C virus
HCV: low molecular mass polypeptide-7
PA28: the proteasome activator complex subunit 1
PAMP: pathogen-associated molecular pattern
ISG15: ISG15
RIG-1: retinoic acid-inducible gene 1
Jak-STAT1: Janus kinase-signal transducer and activator of transcription-1
MAVS: mitochondrial antiviral signaling protein
PP2A: protein phosphatase 2A
PRMT-1: protein arginine N-methyltransferase 1
